# Implications of Social Support as a Self-Control Resource

**DOI:** 10.3389/fnbeh.2016.00228

**Published:** 2016-11-28

**Authors:** June J. Pilcher, Stewart A. Bryant

**Affiliations:** Department of Psychology, Clemson UniversityClemson, SC, USA

**Keywords:** self-control, social support, stress, decision making, goal pursuit, executive functioning

## Abstract

Self-control is an intricate component of decision making and effectively managing day-to-day life. Failing to maintain adequate self-control can have negative effects on many desired goals and social experiences. As such, understanding how different facets of the human experience may affect self-control is an important undertaking. One area that is yet unclear is the possible relationships between social support and self-control. Research suggests that social support can be an effective resource in reducing stress and promoting health and well-being. Research has also indicated that stress can be a limiting factor on self-control. In contrast, few studies have focused on social support as a potential resource for self-control. The goal of this mini-review article is to explore the intersections between self-control and social support and encourage integration of these two relatively independent areas of research. This review will help provide a broader understanding of self-control resources and how we can better understand the relationships between social well-being and our ability to monitor and utilize our capacity to maintain self-control.

## Introduction

Self-control of behavior and emotions is a major component of executive functioning and success in modern society. However, self-control is often negatively affected by events and stressors that are a part of daily life. Stress can result in acute and chronic fatigue which can lead to impulsivity and poor decision making due to a lack of internal resources to maintain self-control (Baumeister et al., 1998; McEwen, 1998). One way to better manage stress could be through social support. Social support has been associated with not only the ability to manage and reduce stress but also to lower the overall perception of stress (Uchino, 2009). Research on the relationship between stress and self-control as well as between stress and social support is growing (Livingston et al., 2015); however, little effort has been made to link social support with self-control. The purpose of this mini-review is to discuss stress, social support and self-control and how they may interact to affect daily functioning.

## Stress

Stress occurs when an individual interprets something as demanding or dangerous causing a negative emotional and alerting response (Baum, 1990). Stressful experiences induce a physiological response resulting from activation of the sympathetic nervous system (SNS) and the hypothalamic-pituitary-adrenocortical (HPA) axis. The HPA response often occurs after an emotional response is elicited from the limbic system and prefrontal cortex (McEwen, 1998; Dickerson and Kemeny, 2004) and results in the release of glucocorticoids. Low concentration of glucocorticoids, particularly cortisol, is necessary for many important cognitive and physiological processes including attention, vasoconstriction, heart rate, and mobility of glucose, protein, and fat (Dickerson and Kemeny, 2004). Under normal conditions, the release of cortisol is healthy but if cortisol is constantly elicited through the HPA axis by chronic stress, damage to the body caused by inflammation can occur. Furthermore, constant release of cortisol flattens diurnal rhythms and creates a reduced physiological response to a perceived stressor (McEwen, 1998).

Because of the over-stimulating nature of today’s society, the SNS is often activated even when not needed. Evolutionarily this SNS response was important for survival, but in the modern age, it can be turned on by events or stimuli that are not actually threatening. Perceptions of threats that are not necessarily warranted (e.g., giving a speech) create excessive release of cortisol (Dickerson and Kemeny, 2004). High levels of corticosteroids reduce inflammation and the ability to fight off foreign pathogens due to its interference with the normal activity of the immune system. As such, the build-up of cortisol over time produces negative effects on the body including immunosuppression, hippocampal atrophy and development of diabetes and cardiovascular disease (Berk et al., 1989; McEwen, 1998; Payne et al., 2002).

One way to cope with high levels of stress is to employ buffering measures that can help alleviate perceived stress (DeLongis and Newth, 1998). Possible buffering measures include adaptive measures (e.g., relaxation, humor, breathing exercises, redefining the situation) and maladaptive measures (e.g., recreational drugs, alcohol, occupational absence, tobacco; Newman and Stone, 1996; Aspinwall and Taylor, 1997). This broad area of buffering measures also encompasses the ability of individuals to properly manage stress through coping and monitoring emotions, behaviors and cognitions related to goal-driven behaviors and self-control.

## Self-Control and Stress

Self-control is the physiological and psychological ability to maintain homeostasis (Baumeister and Vohs, 2007). In particular, it can help explain the focus and ability individuals have to accept delayed gratification in the pursuit of desired goals (Oertig et al., 2013). Self-control includes a complex array of biological and cognitive processes and can be viewed as the multifaceted ability of the individual to control emotions, behaviors and cognitions in a proactive manner to achieve a goal in a given environment (Bandura, 1991; Baumeister et al., 1998; Elliot, 1999; Fitzsimons and Bargh, 2004; Cohen, 2012).

Maintaining control over behavior when pursuing or maintaining a goal comes at the cost of energy and internal willpower used for executive functioning and decision making (Fitzsimons and Bargh, 2004; Oertig et al., 2013). When actively exhibiting self-control, individuals expend internal resources that are believed to be finite (Vohs and Faber, 2007). As such, many researchers contend that the energy cost associated with self-control is drawn from a pool of internal resources that are capable of being exhausted (Aspinwall and Taylor, 1997; Baumeister et al., 1998; Muraven et al., 1998; Muraven and Baumeister, 2000; Vohs and Heatherton, 2000; Baumeister and Vohs, 2007).

One theory of self-control considers the necessary internal resources in terms of a strength model. This model depicts one’s ability to maintain self-control as a metaphorical “muscle” that is capable of fatigue and failure if it is pushed too far (Baumeister et al., 1998; Oertig et al., 2013). When the ability to maintain control is exhausted, the capability to process information and control cognitions, behaviors and emotions efficiently is lost (Oertig et al., 2013). This effect is known as *ego depletion*. Following ego depletion, decision making becomes less efficient, control of emotions is diminished, impulsivity increases, perseverant behavior decreases and goal achievement becomes less important (Baumeister et al., 1998). An individual experiencing ego depletion typically finds it difficult to override or regulate imprudent thoughts, habits or behaviors when necessary. Self-control creates changes in the affect and emotional control, cognitions and behaviors an individual uses to achieve her or his goals. The ability to maintain self-control is intrinsic, but like muscles, the quality of self-control and the quantity capable of being produced before becoming fatigued varies from individual to individual (Cohen, 2012).

Another model of self-control suggests that motivation is the predominant factor in loss of self-control. Inzlicht et al. (2014) maintain that lapses in self-control occur when switching between required and leisure goals. This suggests that self-control is needed when choosing between competing desires or goals. Other researchers have supported this model of self-control and have suggested that self-control could be a matter of correct allocation of effort (Beedie and Lane, 2012) or could result from the person’s belief in internal willpower (Job et al., 2010). Stress researchers have supported a similar approach when emphasizing that recruiting necessary internal resources could result in better performance under stress-inducing conditions, but could also result in other subjective and physiological costs (Hockey, 1997).

To better integrate stress and self-control, it is useful to integrate the two models of self-control. Both models can be used to explain self-control under relatively minor stress conditions (Vohs et al., 2012), suggesting that stress could be a primary factor causing the depletion of resources needed for self-control. Life stressors moderate coping responses by depleting resources necessary for coping with new stressors (Cohen and Lazarus, 1979). Even relatively short stressors have a negative effect on self-control, such as the effects of sleep deprivation on performance (Pilcher et al., 2013), the effects of sleep deprivation on emotional control (Pilcher et al., 2015a), poor sleep habits (Pilcher et al., 2015b), trying to resist multiple temptations (Heatherton and Wagner, 2011), an argument with a loved one or supervisor (Baumeister et al., 1998) and shiftwork (McClelland et al., 2013). Stress also negatively affects goal-oriented behaviors (Hooker et al., 2013). Those who have low self-control ability are more likely to be derailed from goal achievement when stressed compared to individuals with higher self-control ability (Hooker et al., 2013).

## Social Support

Social interactions are one of the most positive and rewarding relationships that humans experience (Krach et al., 2010). Social support is one aspect of social interactions and has been linked with many facets of health, including emotional health, mental health, physical health and well-being (Hefner and Eisenberg, 2009). Social support is the collective structure for help or aid from a mixture of relationships such as friends, family, significant others and acquaintances (Cohen and Wills, 1985). This help can be in the form of perceived social support (the sense of help being available), or received social support (Bolger and Amarel, 2007). In general, higher levels of social support help provide resources not available to those with low levels of social support when seeking help (Cohen and Wills, 1985; Uchino, 2009).

Perceived social support is when individuals believe they have support available from friends, family, significant others, or any individual who would help them when needed (Gottlieb and Bergen, 2010). Research on perceived support has found that when individuals perceive a high level of social support they tend to be healthier and better at coping with stress (Bolger and Amarel, 2007). On the other hand, those who have high levels of received support (actual support provided by others) tend to need help more often, have lower self-esteem, and have trouble coping with stress (Bolger and Amarel, 2007; Uchino, 2009). As such, perceived social support often has a more positive outcome when coping with stressful situations. Unlike perceived support, received support can have negative outcomes when individuals feel like they are unable to help themselves when they feel a need to access help from others (Gottlieb and Bergen, 2010). Moreover, a relationship between received support and depression is present in that the constant need for help is associated with lower self-worth (Barrera, 2000; Liang et al., 2001; Hefner and Eisenberg, 2009). For the purposes of this mini-review, we will focus on the effects of perceived social support.

Social support is divided into two basic models: the main-effect model and the stress-buffering model. According to the main-effect model, social support produces a positive emotional and physical response on the immune and neuroendocrine systems (Cohen and Wills, 1985; House et al., 1988). Having regular social interactions also deters unhealthy behaviors (such as smoking tobacco and alcoholism) while promoting healthy behaviors (such as seeing the doctor) meant to maintain the individual’s well-being (Cohen and Wills, 1985; Uchino, 2009). Although the main-effect model explains how social support can improve well-being and deter unhealthy behaviors, it does not address the functional use of social support.

In contrast, the stress-buffering model views social support as a response tool for deferring or dampening imminent stress (Aspinwall and Taylor, 1997) and as a protective measure against stress. Individuals can be supported before, during and after any stressful event through many different mechanisms including: esteem support, information support, companionship support or instrumental support (Cohen and Wills, 1985; Hefner and Eisenberg, 2009). Each of these areas function to reduce stress with the help of the support group. Esteem support is social support that provides a feeling of self-worth and usefulness to help cope with stress. Information support is support from a person giving advice or talking you through a stressor. Companionship is where close individuals are able to spend recreational time together recuperating from stress. Finally, instrumental support is the use of devices and services an individual can access to aid with the stressor (Cohen and Wills, 1985; Wills, 1991; Uchino, 2009). These elements of social support create different options for preventing stress and coping with stress.

## Social Support and Stress

Social support has been implicated as a buffer of perceived stress in individuals. Those who have more perceived social support are capable of handling stressful or life changing events better than those who lack social support (Cobb, 1976). In addition, those with more social support see stressful situations as more controllable or have less of a stress physiological response due to the additional resources they can draw on to reduce stress (Kirschbaum et al., 1995). Possible ways that perceived social support can buffer stress include preventing the individual from negatively reacting to a stressor by redefining it as not stressful, increasing an individual’s ability to proactively and reactively cope with the stressor, providing supportive solutions for stress, or having an anxiolytic effect on the brain (Cohen and Wills, 1985). Access to social support when under acute stress attenuates free saliva cortisol concentrations and lowers cardiovascular reactivity (Gerin et al., 1992). More broadly in terms of health, perceived social support is negatively correlated with mortality rates due to cardiovascular disease and higher blood pressure (Uchino et al., 1996; Uchino, 2009).

Perceived social support may also be highly correlated with the release of oxytocin. Oxytocin is a neuropeptide that relaxes individuals and is released when they are interacting with others. The amount of oxytocin released is positively correlated with the closeness of the individuals interacting (Heinrichs et al., 2003; Kelly et al., 2012) and creates a calming response for individuals, especially when they are under stress (Heinrichs et al., 2003). Individuals with high social support perceive a stressor as less stressful compared to individuals with low social support. Research suggests that high social support has buffering effects that may be mediated through increased oxytocin concentrations, suggesting that oxytocin may be implicated in the reduction of free cortisol levels that increase during stressful events (Heinrichs et al., 2003).

## Self-Control Resources as a Function of Social Support

Although social support could be a resource for self-control, little research has concentrated on this possible relationship. Social support could be one method to better manage and replenish resources needed for self-control especially under stress-inducing situations. As such, it is possible that stressed individuals with lower social support are less able to cope or effectively self-regulate during stress-inducing situations.

There is limited research that examines possible interactions among social support, self-control and stress. Researchers have concluded that social support is one factor that could influence cognitive appraisal of and, thus, coping with a stressful event (Lazarus and Folkman, 1984). Personality traits could also influence this relationship. Individuals who score high in neuroticism on a trait personality test generally have higher social anxiety and perceive less social support (Arnetz et al., 1985; Uchino, 2009) and are also prone to perceiving more stress in day-to-day activity than those with low neuroticism (Oertig et al., 2013). Duits et al. (1998) found individuals with higher anxiety prior to cardiovascular surgery adjusted worse during their recovery. Conversely, individuals high in extraversion and conscientiousness have more social support, reduced illness, more healthy behaviors and increased longevity even when suffering from chronic illness (Hooker et al., 2013). Furthermore, individuals who attempt to cope with a stressful environment are more likely to engender social support (Schwarzer and Knoll, 2007). Since individuals with higher social support also tend to have more choices for coping with stress than those with low social support, it is possible that a high degree of social support may contribute to self-control resources.

## Implications for Future Research

A complete review of all literature on social support is beyond the capability of this mini-review article; however, the information reviewed here suggests that there could be a three-way relationship among social support, self-control and stress. More targeted research is needed to better identify the possible links, possible moderating variables, and the potential effects on daily coping behaviors, health, and well-being. We have suggested that stress is related to both social support and self-control. Based on earlier literature, especially literature examining coping, it is likely that social support could directly impact the broader construct of self-control. However, very little research has addressed any possible connections, particularly in recent years when research on self-control has started to broaden to examine possible indicators and different measures of self-control. It seems likely that there are multiple pathways to positive self-control outcomes particularly when self-control is required in stress-inducing conditions. Multiple pathways to health-related issues such as heart disease or major depression are well-established. It is feasible that multiple paths are also part of the broader construct of self-control. In this review, we are suggesting that social support could be one of the pathways.

## Conclusions

Maintaining self-control to improve executive functioning in daily life is a constant effort to keep adequate resources available whenever self-control is needed. In modern society, we seem to be constantly challenging our ability to maintain self-control. As such, our energy resources are depleted throughout the day as we attempt to function to the best of our ability. Better understanding how social support may be used to bolster self-control could be a valuable avenue of research to assist individuals with daily functioning.

Currently, there has been little effort to integrate findings from the social support scientific literature with the scientific field of self-control. Integrating these fields could lead to a broader understanding of each field individually and a better understanding of how social support may impact self-control. The possible implications are directly relevant to daily life. Better managing self-control capacity could help with societal issues like addictions, excessive gambling, poor decision making when stressed and over-spending as well as effective executive functioning. In summary, examining the potential impact of social support on self-control both in stress-inducing situations and in non-stressful situations could provide a valued approach to improved daily functioning, health and well-being.

## Author Contributions

JJP and SAB worked together to conceive the concept of a mini-review on this topic. JJP provided oversight for SAB in conducting the literature search and initial review. SAB with input from JJP wrote an early draft of the manuscript. JJP revised and completed the writing of the manuscript. All authors read and approved the final manuscript.

## Funding

The authors have no financial or funding disclosures.

## Conflict of Interest Statement

The authors declare that the research was conducted in the absence of any commercial or financial relationships that could be construed as a potential conflict of interest.
